# Histones participate in base excision repair of 8-oxodGuo by transiently cross-linking with active repair intermediates in nucleosome core particles

**DOI:** 10.1093/nar/gkaa1153

**Published:** 2020-12-08

**Authors:** Mengtian Ren, Mengdi Shang, Huawei Wang, Zhen Xi, Chuanzheng Zhou

**Affiliations:** State Key Laboratory of Elemento-Organic Chemistry and Department of Chemical Biology, College of Chemistry, Nankai University, Tianjin 300071, China; State Key Laboratory of Elemento-Organic Chemistry and Department of Chemical Biology, College of Chemistry, Nankai University, Tianjin 300071, China; State Key Laboratory of Elemento-Organic Chemistry and Department of Chemical Biology, College of Chemistry, Nankai University, Tianjin 300071, China; State Key Laboratory of Elemento-Organic Chemistry and Department of Chemical Biology, College of Chemistry, Nankai University, Tianjin 300071, China; State Key Laboratory of Elemento-Organic Chemistry and Department of Chemical Biology, College of Chemistry, Nankai University, Tianjin 300071, China

## Abstract

8-Oxo-7,8-dihydro-2′-deoxyguanosine (8-oxodGuo) is a biomarker of oxidative DNA damage and can be repaired by hOGG1 and APE1 via the base excision repair (BER) pathway. In this work, we studied coordinated BER of 8-oxodGuo by hOGG1 and APE1 in nucleosome core particles and found that histones transiently formed DNA-protein cross-links (DPCs) with active repair intermediates such as 3′-phospho-α,β-unsaturated aldehyde (PUA) and 5′-deoxyribosephosphate (dRP). The effects of histone participation could be beneficial or deleterious to the BER process, depending on the circumstances. In the absence of APE1, histones enhanced the AP lyase activity of hOGG1 by cross-linking with 3′-PUA. However, the formed histone-PUA DPCs hampered the subsequent repair process. In the presence of APE1, both the AP lyase activity of hOGG1 and the formation of histone-PUA DPCs were suppressed. In this case, histones could catalyse removal of the 5′-dRP by transiently cross-linking with the active intermediate. That is, histones promoted the repair by acting as 5′-dRP lyases. Our findings demonstrate that histones participate in multiple steps of 8-oxodGuo repair in nucleosome core particles, highlighting the diverse roles that histones may play during DNA repair in eukaryotic cells.

## INTRODUCTION

**Figure 1.**
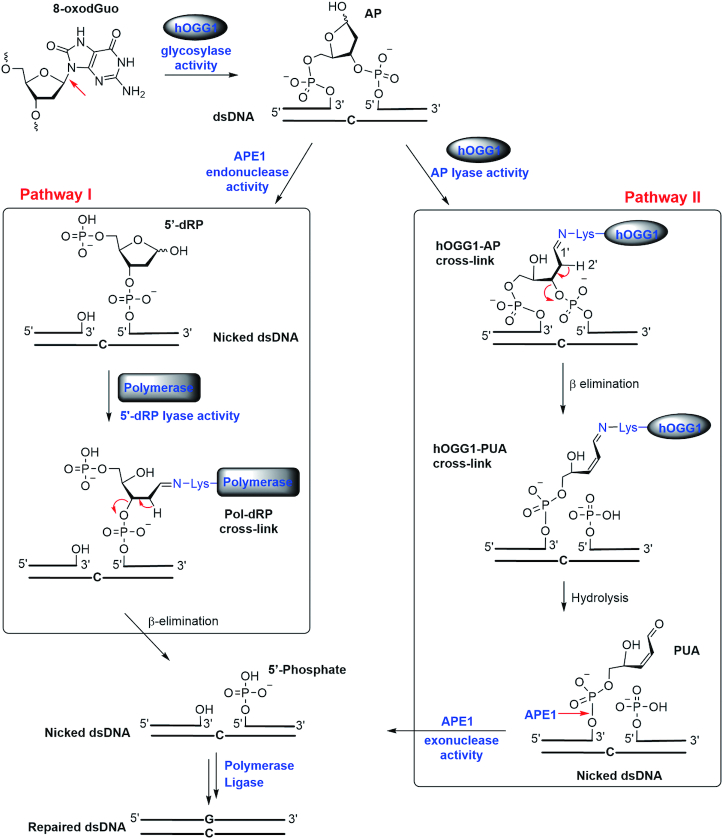
Mechanism of base excision repair of 8-oxodGuo in double-stranded DNA.

8-Oxo-7,8-dihydro-2′-deoxyguanosine (8-oxodGuo) is a common DNA lesion produced under oxidative stress (1,2). To counteract the mutagenic effects of this lesion, cells have evolved efficient repair systems that remove 8-oxodGuo and restore the natural G–C base pair (3). In human cells, the primary repair pathway is base excision repair (BER, Figure 1), which is initiated by hydrolysis of the glycosidic bond by 8-oxoguanine-DNA glycosylase 1 (hOGG1) to generate an abasic site (AP) (4,5). After AP formation, two separate pathways are involved in completion of the repair. In pathway I, the AP is processed by AP endonuclease 1 (APE1), leading to a strand break that generates a 3′-OH and 5′-deoxyribosephosphate (5′-dRP). The 5′-dRP is removed by the 5′-dRP lyase action of a DNA repair polymerase (6,7) to generate a single-nucleotide gap (nicked DNA), which is readily filled by polymerase and ligase. Because hOGG1 is a bifunctional enzyme having both AP lyase and glycosylase activity, the AP can also be processed by a hOGG1-catalysed AP lyase reaction to afford 3′-phospho-α, β-unsaturated aldehyde (PUA) and a 5′-phosphate (pathway II). Subsequently, APE1 acts as an exonuclease to remove the 3′-PUA, which generates the same one-nucleotide gap as pathway I.

In pathway II, the AP lyase activity of hOGG1 is initiated by formation of a C=N linkage between C1′ of the AP and a conserved Lys residue of hOGG1 to generate a species with a DNA-protein cross-link (DPC), designated hOGG1-AP DPC (8). Formation of the C1′=N bond increases the acidity of the 2′-H and thus promotes β-elimination of the 3′-phosphate to give a cleaved DPC intermediate, designated hOGG1-PUA DPC. Spontaneous hydrolysis of the C=N linkage in hOGG1-PUA DPC releases hOGG1, leaving a 3′-PUA terminus. In pathway I, the 5′-dRP lyase activity of polymerase also relies on cross-linking of a Lys residue with an active 5′-dRP, in this case forming Pol-dRP DPC (9,10). Hence, transient cross-linking between active repair intermediates and Lys residues on repair enzymes is extensively employed in DNA repair processes (11).

In eukaryotic cells, double-stranded DNA (dsDNA) is packed in chromatin, the fundamental unit of which is the nucleosome. Nucleosomes are DNA-protein complexes consisting of dsDNA wrapped around an octameric core of histone proteins (12,13). Several recent studies have revealed that the higher-order structure of nucleosomes suppresses the activity of hOGG1 by hindering its access to 8-oxodGuo lesions, and the magnitude of the suppression depends both on the location of the lesion and on the structural dynamics of nucleosomes (14–16).

Histones are Lys-rich, especially in their N-terminal tails (12). Both our group and that of Greenberg have demonstrated that Lys residues of histones in nucleosomes are extensively involved in DNA damage processes (17,18), acting as general acid/base catalysts and/or forming cross-links with active DNA lesions (19–23). Therefore, we hypothesized that histones may also participate in BER by reacting with active repair intermediates such as 3′-PUA and 5′-dRP. In the present work, we studied BER of 8-oxodGuo by hOGG1 and APE1 in nucleosome core particles (NCPs), with a focus on addressing the potential participation of histones. We found that in the absence of APE1, 8-oxodGuo repair occurred via pathway II. Unexpectedly, we found that histone-PUA DPCs were formed in addition to hOGG1-AP and hOGG1-PUA DPCs. The formation of both histone-PUA DPCs and hOGG1-PUA DPC impeded the subsequent repair process. In the presence of APE1, the AP lyase activity of hOGG1 and DPC formation were essentially suppressed in NCPs. Thus, 8-oxodGuo was repaired via pathway I, and after 5′-dRP generation, histones transiently cross-linked with 5′-dRP to catalyse its removal, indicating that histones have 5′-dRP lyase activity.

## MATERIALS AND METHODS

### Materials and general methods

dsDNA and NCPs containing a single 8-oxodGuo/C base pair (referred to as dsDNA-8-oxodGuo and NCP-8-oxodGuo, respectively) at three different positions (73, 89 and 137) were prepared according to the procedure we reported previously (23). dsDNA and NCPs containing a single AP at position 137 (referred to as dsDNA-AP^137^ and NCP-AP^137^, respectively) were obtained by in situ photolysis of dsDNA and NCPs containing a photoprotected AP^137^ (21,24). Unless otherwise specified, dsDNA and NCPs were labelled with fluorescein amidite (FAM) at the 5′ end of the 8-oxodGuo-modified strand. dsDNA and NCPs with a FAM label only on the 3′ end are referred to as dsDNA-8-oxodGuo-3′-FAM and NCP-8-oxodGuo-3′-FAM, respectively. The four histone mutants (H4-del 1−20, H3-del 1−37, H2A-del 1−15, H2B-del 1−31) that we used to prepare tailless NCPs were obtained from Professor Marc Greenberg (Johns Hopkins University). The plasmid used for hOGG1 expression was provided by Professor Bjørn Dalhus (Oslo University Hospital), and hOGG1 was expressed and purified as previously reported (25). APE1 and proteinase K were purchased from NEB (catalogue nos. M0282S and P8107S, respectively). All reactions were carried out in siliconized tubes. Gels were visualized with an Amersham Typhoon Gel and Blot Imaging System at excitation and emission wavelengths of 488 and 526 nm, respectively.

### Repair of 8-oxodGuo by hOGG1 in NCPs

To 50 μl of NCP-8-oxodGuo solution (10 pmol in 10 mM HEPES buffer, pH 7.5, 60 mM NaCl, 0.1 mM PMSF) were added 6 μl of aqueous MgCl_2_ (0.1 M, final concentration 10 mM) and hOGG1 (50 pmol, 5 equiv.). The reaction mixture (total volume 60 μl) was incubated at 37°C, and aliquots were removed periodically. For quantification of the repair efficiency, 1 μl of proteinase K (0.4 unit) and then 1.25 μl of 0.25 M NaOH solution (final concentration 50 mM) were added to each aliquot (4 μl), and the mixture was heated at 70°C for 15 min and then analysed by 8% denaturing PAGE. For monitoring of DPC formation, another aliquot (8 μl) was removed and quenched with NaBH_3_CN (final concentration 50 mM); the resulting sample was analysed by 10% SDS-PAGE.

### Characterization of hOGG1-AP DPC and hOGG1-PUA DPC by denaturing PAGE

To 10 μl of a dsDNA-8-oxodGuo^137^ solution (8 pmol in 10 mM HEPES, pH 7.5, 60 mM NaCl, 0.1 mM PMSF) were added 2 μl of MgCl_2_ (0.1 M, final concentration 10 mM), 4 μl of aqueous NaBH_3_CN (0.25 M, final concentration 50 mM), and hOGG1 (80 pmol). The reaction mixture (total volume 20 μl) was incubated at 37°C for 1 h and then analysed by 10% SDS-PAGE. Another reaction was carried out in the same way except that NaBH_3_CN was added not at the beginning but before 10% SDS-PAGE analysis, to stabilize DPCs. The DPC bands were excised from the SDS-PAGE gel and extracted overnight with elution buffer (0.1% SDS, 0.2 M NaCl and 1 mM EDTA; total volume 500 μl). Ethanol was added to the extract, and the resulting precipitates were dissolved in water (20 μl) and treated with 1 μl of proteinase K (0.8 unit) at room temperature for 10 min. The samples were then analysed by 8% denaturing PAGE.

### Characterization of histone-PUA DPCs by gel shift assay

Authentic histone-PUA DPCs were prepared by means of the following procedure: 2 pmol of dsDNA-AP^137^ was mixed separately with 2 equiv. of a histone (H2A, H2B, H3 or H4) in a siliconized tube (total volume 20 μl), and the mixture was incubated at 37°C for 36 h. Then 5 μl of 0.25 M aqueous NaBH_3_CN (final concentration 50 mM) was added to stabilize the DPCs. Authentic histone-AP DPCs were prepared by means of the procedure used for histone-PUA DPCs, except that NaBH_3_CN was added at the beginning of the reaction.

NCP-8-oxodGuo^137^ was treated with 5 equiv. of hOGG1 at 37°C for 1 h as described above. After quenching with NaBH_3_CN (final concentration 50 mM), the obtained sample was analysed by 10% SDS-PAGE together with the above-described authentic histone-AP DPCs and histone-PUA DPCs.

### Mass spectrometric identification of the histones involved in the histone-PUA DPCs

NCP-8-oxodGuo^137^ (100 pmol, reconstituted in the absence of salmon sperm DNA) was concentrated with an Amicon Ultra centrifugal filter (3K MWCO) at 4°C to a total volume of 40 μl, and then hOGG1 (5 equiv.) and MgCl_2_ (final concentration 10 mM) were added. After the mixture was incubated at 37°C for 2 h, NaBH_3_CN (final concentration 50 mM) was added, and the sample was analysed by 10% SDS-PAGE. The histone-PUA DPC bands were excised, subjected to in-gel tryptic digestion, and analysed by ultra-performance liquid chromatography tandem mass spectrometry, as described in the literature (20).

### Repair of 8-oxodGuo in dsDNA by hOGG1 and APE1 successively

To a solution of dsDNA-8-oxodGuo^137^ (2 pmol in 10 mM HEPES, pH 7.5, 10 mM NaCl, 10 mM MgCl_2_, total volume 20 μl) was added hOGG1 (10 pmol), and the reaction mixture was incubated at 37°C for 10 min. Then APE1 (1 μl, 6 pmol) was added, and incubation was continued at 37°C. Aliquots (4 μl) were removed periodically and quenched with freshly prepared 0.1 M aqueous NaBH_4_ (final concentration 10 mM). Proteinase K (0.5 μl, 0.4 unit) was added to the sample before it was analysed by 8% denaturing PAGE.

### Repair of DPCs by APE1 in NCPs

To 16 μl of a NCP-8-oxodGuo^137^ solution (4 pmol in 10 mM HEPES buffer, pH 7.5, 60 mM NaCl, 0.1 mM PMSF) were added 3.5 μl of MgCl_2_ (0.1 M, final concentration 10 mM) and hOGG1 (20 pmol, 5 equiv.). The reaction mixture was incubated at 37°C for 1 h to allow DPC formation, and then APE1 (12 pmol) was added. Aliquots were removed periodically and quenched with NaBH_3_CN (final concentration 50 mM). The samples were analysed by 10% SDS-PAGE. As a control, dsDNA-8-oxodGuo^137^ was treated in parallel in the same manner, except that the duration of treatment with hOGG1 was reduced to 10 min.

### Repair of 8-oxodGuo by hOGG1 and APE1 in NCPs

To 43 μl of NCP-8-oxodGuo^137^ solution (8 pmol in 10 mM HEPES, pH 7.5, 60 mM NaCl, 0.1 mM PMSF) were added 5 μl of MgCl_2_ (0.1 M, final concentration 10 mM), APE1 (6 pmol), and hOGG1 (8 pmol, 1 equiv.). The reaction mixture was incubated at 37°C, and aliquots were removed periodically. For quantification of repair efficiency by denaturing PAGE, 1 μl of proteinase K (0.4 unit) and then 1 μl of 0.5 M NaOH solution (final concentration 50 mM) were added to an 8 μl aliquot. The resulting mixture was heated at 70°C for 15 min and then analysed by 8% denaturing PAGE. For SDS-PAGE monitoring of DPC formation, 1 μl of 0.25 M aqueous NaBH_3_CN (final concentration 50 mM) was added to a 4 μl aliquot to stabilize the DPCs. These samples were kept at −80°C until analysis by 10% SDS-PAGE.

### Kinetics of 5′-dRP removal

To 28 μl of NCP-8-oxodGuo^137^-3′-FAM solution (10 pmol in 10 mM HEPES, pH 7.5, 60 mM NaCl, 0.1 mM PMSF) were added 5.5 μl of MgCl_2_ (0.1 M, final concentration 10 mM), APE1 (12 pmol) and hOGG1 (50 pmol, 5 equiv.). The reaction mixture was incubated at 37°C, and aliquots were removed periodically. To a 6 μl aliquot were added proteinase K (1 μl, 0.4 unit) and then freshly prepared aqueous NaBH_4_ (final concentration 10 mM), and the resulting mixture was analysed by 8%/20% two-layer denaturing PAGE. Alternatively, the aliquots were quenched with NaBH_3_CN (final concentration 50 mM) and analysed by 15.5% Tricine-SDS-PAGE. As a control, dsDNA-8-oxodGuo^137^-3′-FAM was treated in parallel in the same manner as the samples.

## RESULTS AND DISCUSSION

### Repair of 8-oxodGuo in NCPs by hOGG1 is location dependent

**Figure 2.**
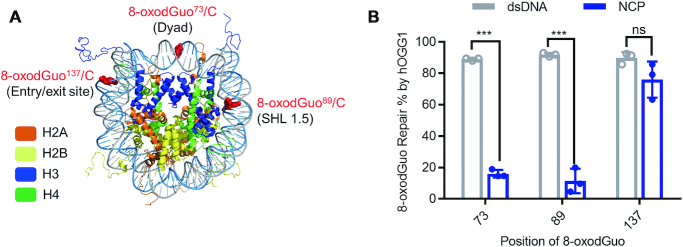
Repair of 8-oxodGuo lesions in NCPs by hOGG1. (**A**) X-ray crystal structure of an NCP (PDB: 1kx5) showing the locations of the 8-oxodGuo/C base pairs. (**B**) Comparison of the 8-oxodGuo repair efficiencies of hOGG1 in NCPs and in free dsDNA. Data are means ± standard deviations of three independent experiments. Statistical significance was determined by means of Student's *t*-test (ns, not significant [*P* > 0.05]; *** *P* < 0.001).

The dsDNA sequence used in this study was 145 bp ‘601’ DNA, which forms stable, well-positioned NCPs (26,27). Following the procedure we reported previously (23), we prepared free dsDNA and NCPs containing a single 8-oxodGuo/C base pair at each of positions 73, 89 and 137 (Figure 2A). To facilitate subsequent gel imaging, we labelled the 8-oxodGuo-modified strand with a 5′-FAM group.

Repair of 8-oxodGuo by hOGG1 generates an AP and other intermediates that undergo quantitative strand cleavage upon treatment with warm NaOH (24). Thus, we incubated 8-oxodGuo-containing free dsDNA and NCPs with excess hOGG1 (5 equiv.) for 2 h and then treated them with NaOH to induce strand cleavage at the position where the 8-oxodGuo had been repaired. The percentages of 8-oxodGuo repair were quantified on the basis of 8% denaturing PAGE results (Supplementary Figure S1). As shown in Figure 2B, nearly 90% of the 8-oxodGuo lesions in the dsDNA were repaired by hOGG1, no matter where they were located.

hOGG1 also repaired 8-oxodGuo lesions in NCPs, but the hOGG1 activity depended strongly on the location of the lesion. The repair percentages for 8-oxodGuo^73^ and 8-oxodGuo^89^ located in the dyad region and at superhelical location (SHL) 1.5 of the NCPs, respectively, were significantly lower than the corresponding percentages in free dsDNA. In contrast, for 8-oxodGuo^137^, which lies at the entry/exit site of the NCPs, the repair percentages were comparable in dsDNA and NCPs. The location-dependent repair of 8-oxodGuo by hOGG1 in NCPs is consistent with previously reported findings (14–16). The relatively higher repair efficiency of 8-oxodGuo at the entry/exit site has been ascribed to transient unwrapping of the DNA from the histone core, which gives hOGG1 easier access to the lesion (16). Because our main research objective was to determine whether histones participate in BER of 8-oxodGuo, the steric effects of NCPs were outside the scope of this work; therefore, we used NCP-8-oxodGuo^137^ as a model system to elucidate the repair mechanism.

### Histones enhance the AP lyase activity of hOGG1 in NCPs by forming histone-PUA DPCs

**Figure 3.**
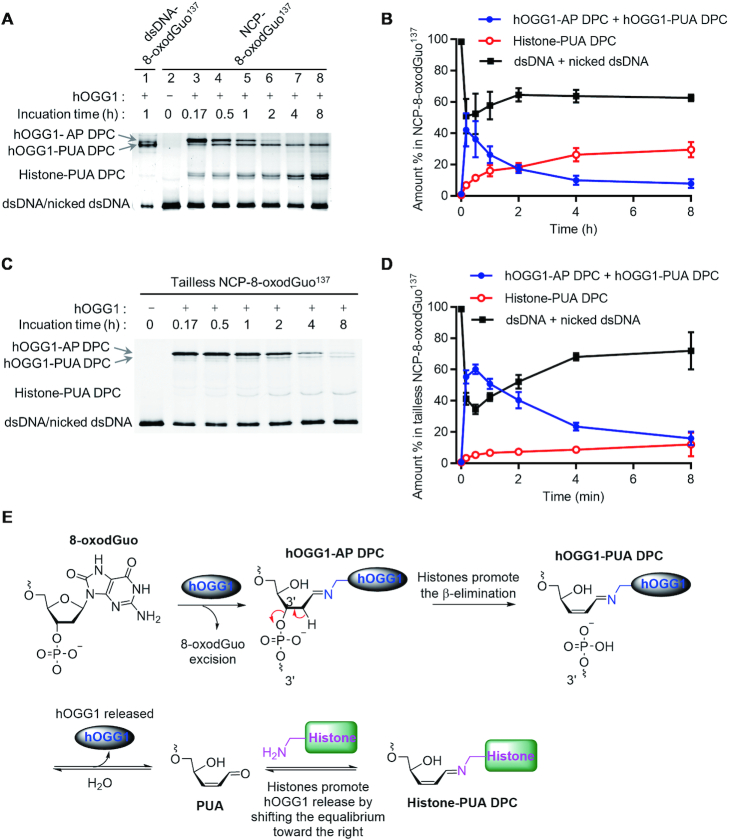
Production of histone-PUA DPCs during 8-oxodGuo repair by hOGG1 in NCPs. (**A** and **C**) 10% SDS-PAGE analyses of 8-oxodGuo^137^ repair by hOGG1 in NCP-8-oxodGuo^137^ and tailless NCP-8-oxodGuo^137^, respectively. (**B** and **D**) Kinetics of 8-oxodGuo^137^ repair by hOGG1 in NCP-8-oxodGuo^137^ and tailless NCP-8-oxodGuo^137^, respectively. (**E**) Proposed mechanism of histone-PUA DPC formation during 8-oxodGuo repair by hOGG1 in NCPs.

In the absence of APE1, hOGG1 shows AP lyase activity after 8-oxodGuo excision from dsDNA. To examine the lyase activity of hOGG1 in NCPs, we treated NCP-8-oxodGuo^137^ with excess hOGG1 and measured the reaction kinetics on the basis of the results of 10% SDS-PAGE. As was the case for dsDNA (lane 1, Figure 3A), two typical hOGG1-DNA cross-linked intermediates were observed during the repair in NCPs (lanes 3–8, Figure 3A). The slower- and faster-migrating DPC species at the top of the gel were identified as hOGG1-AP DPC and hOGG1-PUA DPC, respectively (Supplementary Figure S2). Initially, hOGG1-AP DPC was the predominant product, but the amount of this product decreased rapidly with time, and the decrease was accompanied by an increase in the amount of hOGG1-PUA DPC. These results indicate that hOGG1 also showed AP lyase activity in NCPs in the absence of APE1, leading to strand cleavage via the formation of hOGG1-DNA cross-links.

In addition to hOGG1-AP DPC and hOGG1-PUA DPC, we observed two new products that were not observed during 8-oxodGuo repair by hOGG1 in dsDNA. These new products migrated between hOGG1-PUA DPC and dsDNA (Figure 3A), and treatment with proteinase K transformed them to dsDNA, indicating that they were DPC species as well (Supplementary Figure S2). In this reaction system, histones were the only available proteins other than hOGG1. We previously demonstrated that Lys residues of histones can catalyse AP cleavage in NCPs by forming histone-AP DPC and histone-PUA DPC intermediates (24). Thus, we hypothesized that the two new products were DPC species formed by cross-linking between histones and active repair intermediates. To test this hypothesis, we prepared NCPs containing a single AP at position 137 (NCP-AP^137^) by means of our previously reported strategy (24). Incubation of NCP-AP^137^ in the presence and absence of NaBH_3_CN generated histone-AP DPCs and histone-PUA DPCs, respectively. The new products co-migrated with the authentic histone-PUA DPCs (Supplementary Figure S3). To further characterize the new products, we prepared several histone-AP DPCs and histone-PUA DPCs by mixing dsDNA-AP^137^ with individual histones. A gel shift assay revealed that the two new products co-migrated with histone H3-PUA DPC and histone H4-PUA DPC, respectively; and the slower-migrating product, H3-PUA DPC, was the major product (Supplementary Figures S3 and S4). In-gel tryptic digestion of the two products followed by tandem mass spectrometry also confirmed the presence of histones H3 and H4 (Supplementary Figure S4). These results unambiguously confirmed that histone-PUA DPCs formed during 8-oxodGuo repair by hOGG1 in NCPs.

While the total amount of hOGG1-DNA (hOGG1-AP + hOGG1-PUA) DPCs decreased, the amounts of histone-PUA DPCs increased gradually (Figure 3B); in contrast, the amount of free dsDNA (intact dsDNA + nicked dsDNA) remained almost constant. These results suggest that most of the dsDNA release from the hOGG1-DNA DPCs was transformed to histone-PUA DPCs. After 4 h, the overall amount of hOGG1-DNA DPCs was negligible; and the amounts of histone-PUA DPCs and free dsDNA reached a plateau and remained there for up to 48 h (Supplementary Figure S5). Taken together, these results suggest that the mechanism of 8-oxodGuo repair by hOGG1 in NCPs is that shown in Figure 3E. First, hOGG1 excises the 8-oxodGuo and then catalyses strand cleavage via an AP lyase reaction. During this process, hOGG1-AP DPC and hOGG1-PUA DPC are formed as intermediates. After hOGG1 is released from hOGG1-PUA DPC, the resulting active intermediate 3′-PUA is trapped by Lys residues of histones to form histone-PUA DPCs, which exist in dynamic equilibrium.

Lys residues in the flexible N-terminal tails of histones are probably involved in histone-PUA DPC formation. We therefore prepared tailless NCP-8-oxodGuo^137^ species in which the histones were replaced by four tailless histone mutants (H4-del 1−20, H3-del 1−37, H2A-del 1−15, and H2B-del 1−31), and we analysed the repair kinetics of these tailless species (Figure 3C, D). Comparison with wild-type NCP-8-oxodGuo^137^ revealed three obvious differences: first, the transformation of hOGG1-AP DPC to hOGG1-PUA DPC via β-elimination was much slower in tailless NCP-8-oxodGuo^137^ (Figure 3C); second, the decomposition of all the hOGG1-DNA DPCs to release hOGG1 was slower, and thus hOGG1-DNA DPCs accumulated to a higher level in tailless NCP-8-oxodGuo^137^ (60%) than in WT NCPs (40%) at the beginning of incubation and persisted longer; third, the formation of histone-PUA DPCs was markedly suppressed (8% versus 29% in WT NCPs at equilibrium). These results confirm that the N-terminal tails of histones were the major domains involved in histone-PUA DPC formation. More strikingly, in addition to forming DPCs, histone tails promoted both the β-elimination of 3′-phosphate from hOGG1-AP DPC and the release of hOGG1 from hOGG1-PUA DPC (Figure 3E). The former role is probably attributable to the basic environment provided by the N-terminal tails of histones (28); that is, histones acted as general acid/base catalysts (24,29). The latter role involved trapping of 3′-PUA by histones, thus preventing retrograde reaction of hOGG1 with the active intermediate. Taken together, our results indicate that the Lys-rich, flexible N-terminal tails of histones enhanced the AP lyase activity of hOGG1 in NCPs and that histone-PUA DPC formation was a unique process during 8-oxodGuo repair by hOGG1 in NCPs and contributed to the enhanced AP lyase activity.

### Formation of hOGG1-PUA DPC and histone-PUA DPCs hampers subsequent repair by APE1

After 8-oxodGuo excision from dsDNA and a subsequent hOGG1-catalysed AP lyase reaction, the resulting 3′-PUA terminus is recognized and removed by APE1 (pathway II in Figure 1). Having elucidated the effect of histone-PUA DPC formation on the AP lyase activity of hOGG1, we next investigated the effect of DPC formation on the repair activity of APE1.

**Figure 4.**
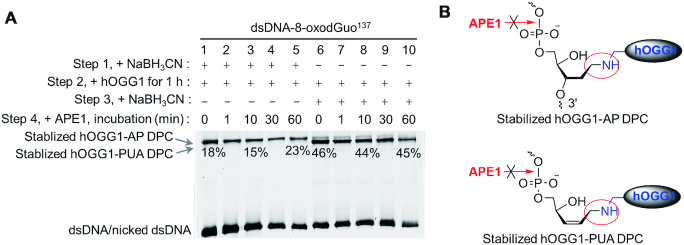
Neither stabilized hOGG1-AP DPC nor stabilized hOGG1-PUA DPC could be repaired by APE1 in dsDNA. (**A**) 10% SDS-PAGE analysis of the repair of stabilized hOGG1-AP DPC and stabilized hOGG1-PUA DPC by APE1 in free dsDNA. (**B**) Structures of stabilized hOGG1-AP DPC and stabilized hOGG1-PUA DPC.

We began by incubating dsDNA-8-oxodGuo^137^ for 1 h with hOGG1 in the presence of NaBH_3_CN, which can selectively reduce the C=N bond to a stable C–N bond and thus transform hOGG1-AP DPC to stabilized hOGG1-AP DPC (Figure 4). As a result, we trapped stabilized hOGG1-AP DPC in 18% yield (lane 1 in Figure 4A). Then APE1 was added to the reaction mixture, and incubation was continued; during this period, the amount of stabilized hOGG1-AP DPC remained constant. In contrast, treating dsDNA-8-oxodGuo^137^ with hOGG1 for 1 h and then adding NaBH_3_CN allowed us to selectively trap stabilized hOGG1-PUA DPC in 46% yield (lane 6 in Figure 4A). Addition of APE1 to the reaction mixture also had no effect on the amount of stabilized hOGG1-PUA DPC. In short, APE1 could not process either stabilized hOGG1-AP DPC or stabilized hOGG1-PUA DPC.

**Figure 5.**
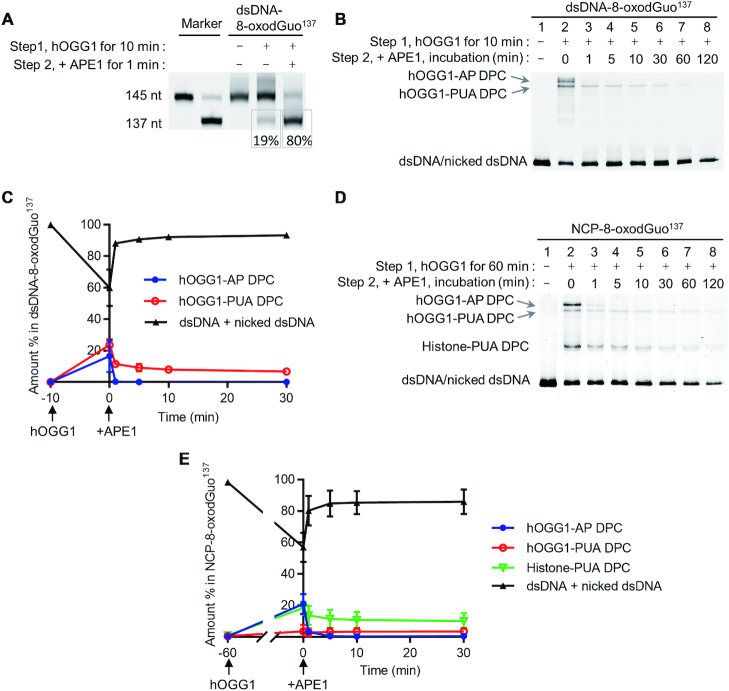
Kinetics of DPC repair by APE1. (**A**) 8% denaturing PAGE analyses of dsDNA-8-oxodGuo^137^ repair by successive treatment with hOGG1 and APE1. (**B** and **D**) 10% SDS-PAGE analyses of DPC repair by APE1 in dsDNA-8-oxodGuo^137^ and NCP-8-oxodGuo^137^, respectively. (**C** and **E**) Kinetics of DPC repair by APE1 in dsDNA-8-oxodGuo^137^ and NCP-8-oxodGuo^137^, respectively.

To examine the ability of APE1 to process real DPCs that formed during 8-oxodGuo repair by hOGG1, we treated dsDNA-8-oxodGuo^137^ successively with hOGG1 and APE1 in the absence of NaBH_3_CN. After protein removal by treatment with proteinase K, 8% denaturing PAGE analysis revealed that treating dsDNA-8-oxodGuo^137^ with hOGG1 for 10 min led to 19% strand cleavage, but subsequent treatment with APE1 led to 80% strand cleavage (Figure 5A). This finding indicates that incubation with hOGG1 for 10 min resulted in excision of more than 80% of 8-oxodGuo but that the main products were an AP and hOGG1-AP DPC, both of which appeared as intact strands (145 nt) in the denaturing gel.

Next we analysed the kinetics of hOGG1-AP DPC and hOGG1-PUA DPC processing by APE1 on the basis of 10% SDS-PAGE data. After treatment of dsDNA-8-oxodGuo^137^ with hOGG1 for 10 min, hOGG1-AP DPC and hOGG1-PUA DPC were obtained in 17% and 23% yields, respectively (lane 2 in Figure 5B, C). When APE1 was added to the reaction mixture and incubation was continued, hOGG1-AP DPC decomposed rapidly and was undetectable after 1 min. However, hOGG1-PUA DPC decomposed much more slowly and was still detectable (3%) after 1 h of incubation (Figure 5B, C).

**Figure 6.**
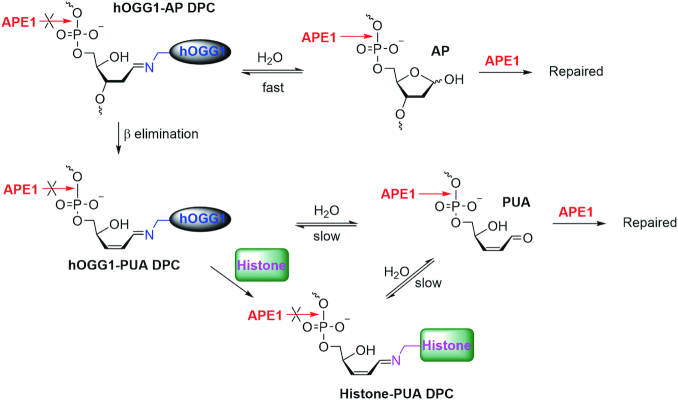
Proposed mechanism for DPC repair by APE1.

A similar trend was observed for NCPs. After treatment of NCP-8-oxodGuo^137^ with hOGG1 for 60 min, addition of APE1 led to rapid decomposition of hOGG1-AP DPC (Figure 5D, E), whereas both hOGG1-PUA DPC and histone-PUA DPCs persisted much longer. Collectively, these results suggest that APE1 could process all the DPCs that formed during 8-oxodGuo repair by hOGG1 in NCPs but that the activities of APE1 toward different types of DPCs were different. Compared with hOGG1-PUA DPC and histone-PUA DPCs, hOGG1-AP DPC was more prone to processing by APE1. This observation seems to contradict the above-described result that neither stabilized hOGG1-AP DPC nor stabilized hOGG1-PUA DPC could be processed by APE1. One reasonable explanation for this contradiction is depicted in Figure 6. hOGG1-AP DPC is not a substrate of APE1, but because hOGG1-AP DPC formation is reversible, once an AP is regenerated by hydrolysis, it can be recognized and incised efficiently by APE1. Because the equilibrium between hOGG1-AP DPC and AP is highly dynamic (24,30), hOGG1-AP DPC appears to be processed by APE1 to yield the repaired product. hOGG1-PUA DPC and histone-PUA DPCs are not substrates of APE1 either, but their hydrolysis produces 3′-PUA, which is a native substrate of APE1 and can be efficiently repaired. Hydrolyses of the α,β-unsaturated C=N bonds of hOGG1-PUA DPC and histone-PUA DPCs to release 3′-PUA are thought to be much slower, resulting in slower apparent kinetics for the repair of hOGG1-PUA DPC and histone-PUA DPCs. Therefore, neither hOGG1-DNA DPCs nor histone-PUA DPCs are native substrates for APE1, and they must be transformed to AP and 3′-PUA before they can be repaired by APE1. Thus, formation of hOGG1-DNA DPC and histone-PUA DPCs during repair by hOGG1 is detrimental to the BER process, which also explains why the hOGG1-catalysed AP lyase reaction is inefficient for BER repair of 8-oxodGuo.

### APE1 stimulates hOGG1 by suppressing DPC formation in NCPs

**Figure 7.**
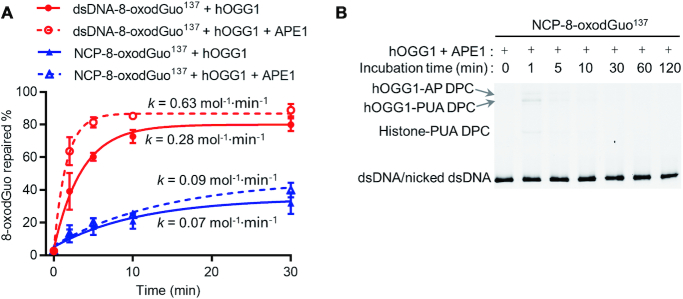
APE1 stimulation of hOGG1 by suppression of DPC formation in NCPs. (**A**) Kinetics of dsDNA-8-oxodGuo^137^ and NCP-8-oxodGuo^137^ repair by hOGG1 in the presence and absence of APE1. (**B**) 10% SDS-PAGE analysis of NCP-8-oxodGuo^137^ repair by hOGG1 in the presence of APE1.

In dsDNA, APE1 can stimulate the turnover of hOGG1 by promoting its release from the product, thus bypassing its inefficient AP lyase activity (30–32). We observed this stimulation effect of APE1 when we used dsDNA-8-oxodGuo^137^ as the substrate. Specifically, when dsDNA-8-oxodGuo^137^ was treated with a mixture of hOGG1 and APE1, the repair was 2 times as fast as that with hOGG1 alone (Figure 7A and Supplementary Figure S6). Under the same conditions, stimulation of hOGG1 by APE1 was also observed in NCP-8-oxodGuo^137^, although the stimulatory effect was much smaller than that observed in dsDNA. To shed light on the origin of the stimulatory effect of APE1 in NCPs, we used 10% SDS-PAGE to analyse the kinetics of NCP-8-oxodGuo^137^ repair with a mixture of hOGG1 and APE1 (Figure 7B). In this experiment, a very small amount of hOGG1-DNA DPC was observed at the beginning of the reaction (1 min), and then it disappeared rapidly. Histone-PUA DPC formation was negligible throughout the whole process. Thus, the presence of APE1 suppressed the formation of both hOGG1-DNA and histone-PUA DPCs. That is, as was the case for free dsDNA, APE1 stimulated hOGG1 by bypassing the AP lyase activity and suppressing DPC formation in NCPs.

In the presence of APE1 and hOGG1, the AP generated in NCPs can be processed by APE1 endonuclease activity, hOGG1 lyase activity, or histone lyase activity (24). The substantial decrease in both hOGG1-DNA DPC and histone-DNA DPC formation upon addition of APE1 suggests that APE1 is more potent than both hOGG1 and histones in AP processing. APE1 is known to be one of the fastest BER enzymes, and it cleaves APs ∼10^3^ times as fast as hOGG1 (33). In contrast, the activities of APE1 and histones for AP processing in NCPs have not been compared. To make this comparison, we generated NCP-AP^137^ in the presence and absence of APE1 (Supplementary Figure S7) and found that APE1-catalzyed strand cleavage at AP^137^ was 10^6^ times as fast as that by histones (in the absence of APE1). That is, both hOGG1 and histones were markedly less efficient than APE1 for AP processing; therefore, bypassing the AP lyase activities of hOGG1 and histones by APE1 favoured a higher overall BER efficiency.

Recently, Pederson *et al.* studied the coordinated repair of thymine glycol by APE1 and hNTHL1, another bifunctional glycosylase (34), and found that in nucleosomes, unlike in free dsDNA, APE1 cannot stimulate hNTHL1 by bypassing the AP lyase activity of hNTHL1. Therefore, stimulation of bifunctional glycosylase by APE1 is common in free dsDNA, whereas in nucleosomes, the stimulation effect of APE1 seems to depend on a specific glycosylase.

### Histones exhibit 5′-dRP lyase activity during BER in NCPs

**Figure 8.**
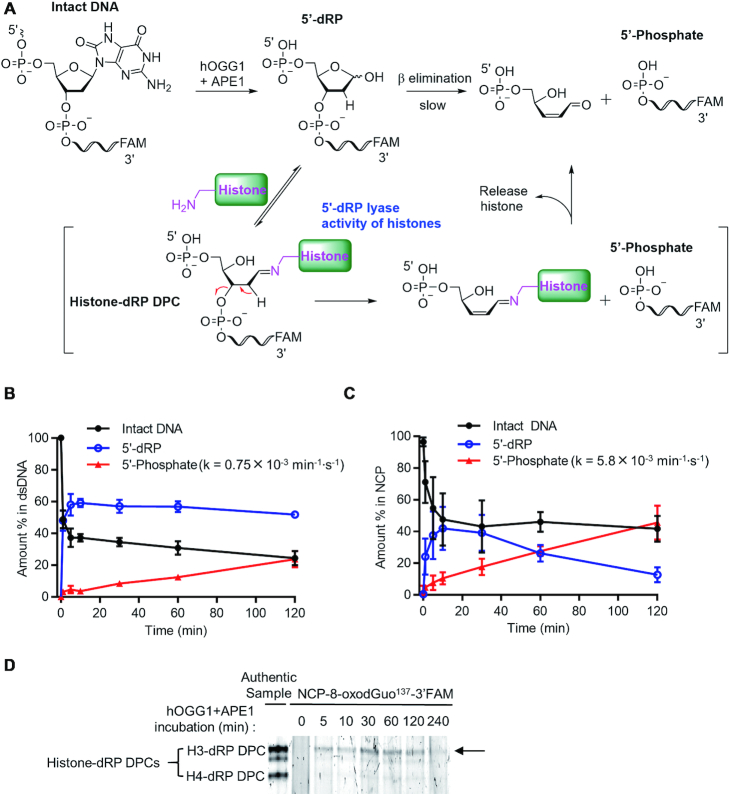
Histone 5′-dRP lyase activity during BER of 8-oxodGuo by hOGG1 and APE1 in NCPs. (**A**) Mechanism of histone-promoted elimination of 5′-dRP. (**B**) and (**C**) Kinetics of repair of dsDNA-8-oxodGuo^137^-3′-FAM and NCP-8-oxodGuo^137^-3′-FAM, respectively, by hOGG1 and APE1. (**D**) 15.5% Tricine-SDS-PAGE analysis of NCP-8-oxodGuo^137^-3′-FAM repair by hOGG1 and APE1.

No matter what repair pathway is followed, BER of 8-oxodGuo by hOGG1 and APE1 eventually leads to a strand break leaving a 3′-OH and 5′-dRP (Figure 1). 5′-dRP can be efficiently removed by a 5′-dRP lyase reaction catalysed by polymerase both in free dsDNA and in nucleosomes (35). In the absence of polymerase, spontaneous β-elimination of 5′-dRP also occurs, generating a 5′-phosphate group (Figure 8A) (36). When we used dsDNA-8-oxodGuo^137^-3′-FAM as the substrate, we observed the slow decomposition of 5′-dRP to generate a 5′-phosphate after treatment with hOGG1 and APE1 (Figure 8B and Supplementary Figure S8). Treatment of NCP-8-oxodGuo^137^-3′-FAM under the same conditions led to a 7-fold increase in the rate of transformation of 5′-dRP to a 5′-phosphate (Figure 8C), indicating that this transformation was markedly promoted in NCPs.

An increased rate of 5′-dRP loss in NCPs was also observed by Wilson and colleagues, who attributed this result to the catalytic effect of the basic environment provided by histones (35). We hypothesized that Lys-rich histones may also catalyse the elimination of 5′-dRP by acting as 5′-dRP lyases (Figure 8A). In this process, histone Lys residues react with the active repair intermediate 5′-dRP to afford histone-dRP DPCs, through which the elimination of 5′-dRP is promoted. To test this hypothesis, we used 10% SDS-PAGE to monitor the kinetics of NCP-8-oxodGuo^137^-3′-FAM repair by hOGG1 and APE1. We did observe a DPC intermediate, the amount of which increased at the beginning of incubation and then decreased with increasing incubation time (Figure 8D). By incubating dsDNA-8-oxodGuo^137^-3′-FAM with hOGG1 and APE1 in the presence of individual histones, we prepared four different histone-dRP DPCs (Supplementary Figure S9). A gel shift assay showed that the DPC intermediate obtained in NCPs was H3-dRP DPC (Figure 8D), which is consistent with the fact that the N-terminal tail of H3 is close to position 137 in NCPs. Taken together, these results confirm that histones have 5′-dRP lyase activity and can catalyse the elimination of 5′-dRP during BER repair in NCPs.

## CONCLUSION

In this work, we studied BER of 8-oxodGuo by hOGG1 and APE1 in NCPs. As in free dsDNA, in NCPs, hOGG1 exhibited both glycosylase activity and AP lyase activity when APE1 was absent. The histones present in NCPs did not bypass the AP lyase activity of hOGG1 but rather enhanced it by playing multiple roles. First, histones promoted β-elimination of 3′-phosphate from hOGG1-AP DPC to generate hOGG1-PUA DPC; second, histones promoted the release of hOGG1 from hOGG1-PUA DPC by forming histone-PUA DPCs. However, the formation of hOGG1-PUA DPC and histone-PUA DPCs hampered subsequent repair by APE1. Therefore, histones inhibited BER of 8-oxodGuo in NCPs in the absence of APE1.

In the presence of APE1, the lyase activity of hOGG1 was remarkably suppressed in NCPs, and histone-PUA DPC formation was negligible. Thus, APE1 could stimulate hOGG1 by bypassing its lyase activity not only in free dsDNA but also in NCPs. After coordinated BER of 8-oxodGuo by hOGG1 and APE1 in NCPs, accelerated loss of 5′-dRP was observed. We successfully trapped the histone-dRP DPC intermediates, indicating that histones could catalyse the loss of 5′-dRP by acting as 5′-dRP lyases. To our knowledge, this is the first demonstration that histones have 5′-dRP lyase activity.

Taken together, our findings indicate that histones exert different effects during different repair steps by transiently cross-linking with active repair intermediates. It is worth noting that BER of 8-oxodGuo is a multistep process and that hOGG1, APE1 and other repair enzymes, such as polymerase and ligase, act in a coordinated manner to complete the repair (33,37). Therefore, an effect that is beneficial in one step can be deleterious to the overall process, and vice versa. For instance, we demonstrated that histones play a beneficial role during 5′-dRP removal by acting as 5′-dRP lyases. However, if a DNA repair polymerase is present, 5′-dRP may be removed more efficiently by the 5′-dRP lyase action of the polymerase. That is, the 5′-dRP lyase activity of histones can be detrimental to BER of 8-oxodGuo overall. For another example, although histone-PUA DPC formation seems deleterious, because it hampers subsequent repair by APE1, DPC formation may be favourable at double strand breaks. In this situation, DPC formation prevent the broken strands from separating, which leaves enough time for APE1 to find the lesion. Therefore, estimating the overall impact of histones on BER efficiency is difficult. Studying the repair processes in the presence of all the relevant enzymes may provide valuable information and will be the focus of future research.

## Supplementary Material

gkaa1153_Supplemental_File

## References

[1] Cadet J., Douki T., Ravanat J.L. Oxidatively generated damage to the guanine moiety of DNA: mechanistic aspects and formation in cells. Acc. Chem. Res. 2008; 41:1075–1083. 18666785 10.1021/ar700245e

[2] Kasai H. Analysis of a form of oxidative DNA damage, 8-hydroxy-2'-deoxyguanosine, as a marker of cellular oxidative stress during carcinogenesis. Mutat. Res. 1997; 387:147–163. 9439711 10.1016/s1383-5742(97)00035-5

[3] David S.S., O'Shea V.L., Kundu S. Base-excision repair of oxidative DNA damage. Nature. 2007; 447:941–950. 17581577 10.1038/nature05978 PMC2896554

[4] Rogacheva M.V., Kuznetsova S.A. Repair of 8-oxoguanine in DNA. The mechanisms of enzymatic catalysis. Russ. Chem. Rev. 2008; 77:817–843.

[5] Hazra T.K., Das A., Das S., Choudhury S., Kow Y.W., Roy R. Oxidative DNA damage repair in mammalian cells: A new perspective. DNA Repair (Amst.). 2007; 6:470–480. 17116430 10.1016/j.dnarep.2006.10.011 PMC2702509

[6] Allinson S.L., Dianova I.I., Dianov G.L. DNA polymerase β is the major dRP lyase involved in repair of oxidative base lesions in DNA by mammalian cell extracts. EMBO J. 2001; 20:6919–6926. 11726527 10.1093/emboj/20.23.6919 PMC125762

[7] Bebenek K., Tissier A., Frank E.G., McDonald J.P., Prasad R., Wilson S.H., Woodgate R., Kunkel T.A. 5'-Deoxyribose phosphate lyase activity of human DNA polymerase ι in Vitro. Science. 2001; 291:2156–2159. 11251121 10.1126/science.1058386

[8] Nash H.M., Lu R., Lane W.S., Verdine G.L. The critical active-site amine of the human 8-oxoguanine DNA glycosylase, hOgg1: direct identification, ablation and chemical reconstitution. Chem. Biol. 1997; 4:693–702. 9331411 10.1016/s1074-5521(97)90225-8

[9] García-Díaz M., Bebenek K., Kunkel T.A., Blanco L. Identification of an intrinsic 5′-deoxyribose-5-phosphate lyase activity in human DNA polymerase λ: A possible role in base excision repair. J. Biol. Chem. 2001; 276:34659–34663. 11457865 10.1074/jbc.M106336200

[10] Prasad R., Longley M.J., Sharief F.S., Hou E.W., Copeland W.C., Wilson S.H. Human DNA polymerase θ possesses 5′-dRP lyase activity and functions in single-nucleotide base excision repair in vitro. Nucleic Acids Res. 2009; 37:1868–1877. 19188258 10.1093/nar/gkp035 PMC2665223

[11] Tretyakova N.Y., Groehler A., Ji S. DNA–protein cross-links: Formation, structural identities, and biological outcomes. Acc. Chem. Res. 2015; 48:1631–1644. 26032357 10.1021/acs.accounts.5b00056 PMC4704791

[12] McGinty R.K., Tan S. Nucleosome structure and function. Chem. Rev. 2015; 115:2255–2273. 25495456 10.1021/cr500373h PMC4378457

[13] Luger K., Mader A.W., Richmond R.K., Sargent D.F., Richmond T.J. Crystal structure of the nucleosome core particle at 2.8 A resolution. Nature. 1997; 389:251–260. 9305837 10.1038/38444

[14] Menoni H., Shukla M.S., Gerson V., Dimitrov S., Angelov D. Base excision repair of 8-oxoG in dinucleosomes. Nucleic Acids Res. 2012; 40:692–700. 21930508 10.1093/nar/gkr761 PMC3258150

[15] Bilotti K., Kennedy E.E., Li C., Delaney S. Human OGG1 activity in nucleosomes is facilitated by transient unwrapping of DNA and is influenced by the local histone environment. DNA Repair (Amst.). 2017; 59:1–8. 28892740 10.1016/j.dnarep.2017.08.010 PMC5643252

[16] Bilotti K., Tarantino M.E., Delaney S. Human oxoguanine glycosylase 1 removes solution accessible 8-oxo-7,8-dihydroguanine lesions from globally substituted nucleosomes except in the dyad region. Biochemistry. 2018; 57:1436–1439. 29341606 10.1021/acs.biochem.7b01125

[17] Sczepanski J.T., Wong R.S., McKnight J.N., Bowman G.D., Greenberg M.M. Rapid DNA-protein cross-linking and strand scission by an abasic site in a nucleosome core particle. Proc. Natl. Acad. Sci. U.S.A. 2010; 107:22475–22480. 21149689 10.1073/pnas.1012860108 PMC3012510

[18] Ren M., Bai J., Xi Z., Zhou C. DNA damage in nucleosomes. Sci. China Chem. 2019; 62:561–570.

[19] Zhou C.Z., Sczepanski J.T., Greenberg M.M. Histone modification via rapid cleavage of C4 '-oxidized abasic sites in nucleosome core particles. J. Am. Chem. Soc. 2013; 135:5274–5277. 23531104 10.1021/ja400915w PMC3638250

[20] Li F., Zhang Y., Bai J., Greenberg M.M., Xi Z., Zhou C. 5-Formylcytosine yields DNA–protein cross-links in nucleosome core particles. J. Am. Chem. Soc. 2017; 139:10617–10620. 28742335 10.1021/jacs.7b05495 PMC5649621

[21] Shang M., Ren M., Zhou C. Nitrogen mustard induces formation of DNA–histone cross-links in nucleosome core particles. Chem. Res. Toxicol. 2019; 32:2517–2525. 31726825 10.1021/acs.chemrestox.9b00354

[22] Ren M., Cheng Y., Duan Q., Zhou C. Transesterification reaction and the repair of embedded ribonucleotides in DNA are suppressed upon the assembly of DNA into nucleosome core particles. Chem. Res. Toxicol. 2019; 32:926–934. 30990021 10.1021/acs.chemrestox.9b00059

[23] Bai J., Zhang Y., Xi Z., Greenberg M.M., Zhou C. Oxidation of 8-oxo-7,8-dihydro-2′-deoxyguanosine leads to substantial DNA-histone cross-links within nucleosome core particles. Chem. Res. Toxicol. 2018; 31:1364–1372. 30412392 10.1021/acs.chemrestox.8b00244 PMC6425731

[24] Zhou C.Z., Sczepanski J.T., Greenberg M.M. Mechanistic studies on histone catalyzed cleavage of apyrimidinic/apurinic sites in nucleosome core particles. J. Am. Chem. Soc. 2012; 134:16734–16741. 23020793 10.1021/ja306858m PMC3477373

[25] Dalhus B., Forsbring M., Helle I.H., Vik E.S., Forstrøm R.J., Backe P.H., Alseth I., Bjørås M. Separation-of-function mutants unravel the dual-reaction mode of human 8-oxoguanine DNA glycosylase. Structure. 2011; 19:117–127. 21220122 10.1016/j.str.2010.09.023

[26] Vasudevan D., Chua E.Y.D., Davey C.A. Crystal structures of nucleosome core particles containing the '601' strong positioning sequence. J. Mol. Biol. 2010; 403:1–10. 20800598 10.1016/j.jmb.2010.08.039

[27] Lowary P.T., Widom J. New DNA sequence rules for high affinity binding to histone octamer and sequence-directed nucleosome positioning. J. Mol. Biol. 1998; 276:19–42. 9514715 10.1006/jmbi.1997.1494

[28] Yang K., Greenberg M.M. Histone tail sequences balance their role in genetic regulation and the need to protect DNA against destruction in nucleosome core particles containing abasic sites. ChemBioChem. 2019; 20:78–82. 30307690 10.1002/cbic.201800559 PMC6317330

[29] Zhou C.Z., Greenberg M.M. Histone-catalyzed cleavage of nucleosomal DNA containing 2-deoxyribonolactone. J. Am. Chem. Soc. 2012; 134:8090–8093. 22551239 10.1021/ja302993h PMC3354019

[30] Esadze A., Rodriguez G., Cravens S.L., Stivers J.T. AP-endonuclease 1 accelerates turnover of human 8-oxoguanine DNA glycosylase by preventing retrograde binding to the abasic-site product. Biochemistry. 2017; 56:1974–1986. 28345889 10.1021/acs.biochem.7b00017 PMC5526596

[31] Vidal A.E., Hickson I.D., Boiteux S., Radicella J.P. Mechanism of stimulation of the DNA glycosylase activity of hOGG1 by the major human AP endonuclease: bypass of the AP lyase activity step. Nucleic Acids Res. 2001; 29:1285–1292. 11238994 10.1093/nar/29.6.1285 PMC29755

[32] Hill J.W., Hazra T.K., Izumi T., Mitra S. Stimulation of human 8-oxoguanine-DNA glycosylase by AP-endonuclease: potential coordination of the initial steps in base excision repair. Nucleic Acids Res. 2001; 29:430–438. 11139613 10.1093/nar/29.2.430 PMC29662

[33] Schermerhorn K.M., Delaney S. A chemical and kinetic perspective on base excision repair of DNA. Acc. Chem. Res. 2014; 47:1238–1246. 24646203 10.1021/ar400275a PMC3993943

[34] Maher R.L., Wallace S.S., Pederson D.S. The lyase activity of bifunctional DNA glycosylases and the 3′-diesterase activity of APE1 contribute to the repair of oxidized bases in nucleosomes. Nucleic Acids Res. 2019; 47:2922–2931. 30649547 10.1093/nar/gky1315 PMC6451105

[35] Rodriguez Y., Howard M.J., Cuneo M.J., Prasad R., Wilson S.H. Unencumbered Pol β lyase activity in nucleosome core particles. Nucleic Acids Res. 2017; 45:8901–8915. 28911106 10.1093/nar/gkx593 PMC5587807

[36] Admiraal S.J., O’Brien P.J. Reactivity and cross-linking of 5′-terminal abasic sites within DNA. Chem. Res. Toxicol. 2017; 30:1317–1326. 28485930 10.1021/acs.chemrestox.7b00057 PMC5564299

[37] Pascucci B., Maga G., Hubscher U., Bjoras M., Seeberg E., Hickson I.D., Villani G., Giordano C., Cellai L., Dogliotti E. Reconstitution of the base excision repair pathway for 7,8-dihydro-8-oxoguanine with purified human proteins. Nucleic Acids Res. 2002; 30:2124–2130. 12000832 10.1093/nar/30.10.2124 PMC115284

