# Supplementary material for: Histones participate in base excision repair of 8-oxodGuo by transiently cross-linking with active repair intermediates in nucleosome core particles
Source: Nucleic Acids Res. 2020 Dec 8;49(1):257–68. doi: 10.1093/nar/gkaa1153 (PMC7797075; doi:10.1093/nar/gkaa1153)
Supplement: gkaa1153_Supplemental_File [file gkaa1153_supplemental_file.pdf]

SUPPLEMENTARY INFORMATION

Histones Participate in Base Excision Repair of 8-oxodGuo by Transiently Cross-Linking with Active Repair Intermediates in Nucleosome Core Particles

Mengtian Ren, Mengdi Shang, Huawei Wang, Zhen Xi, and Chuanzheng Zhou*

State Key Laboratory of Elemento-Organic Chemistry and Department of Chemical Biology, College of Chemistry, Nankai University, Tianjin 300071, China

* Corresponding author: Chuanzheng Zhou. Email: chuanzheng.zhou@nankai.edu.cn

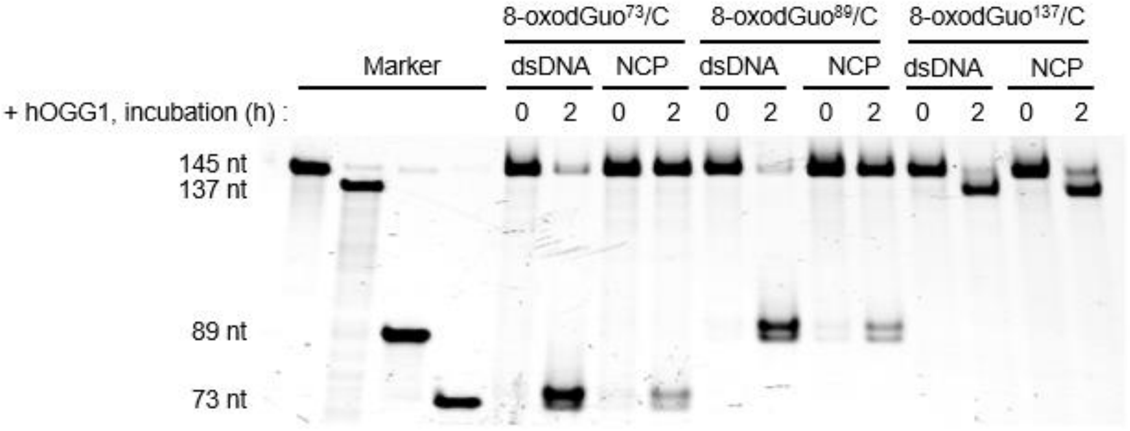

Supplementary Figure S1. Denaturing PAGE (8%) analyses of hOGG1 repair of dsDNA or NCPs containing a single 8-oxodGuo/C base pair at different positions. dsDNA and NCPs containing a single 8-oxodGuo/C base pair at different positions were treated with excess hOGG1 (5 equiv.) for 2 h. The obtained samples are treated with 50 mM NaOH at 70 °C for 15 min to induce strand cleavage at the repaired 8-oxodGuo positions.

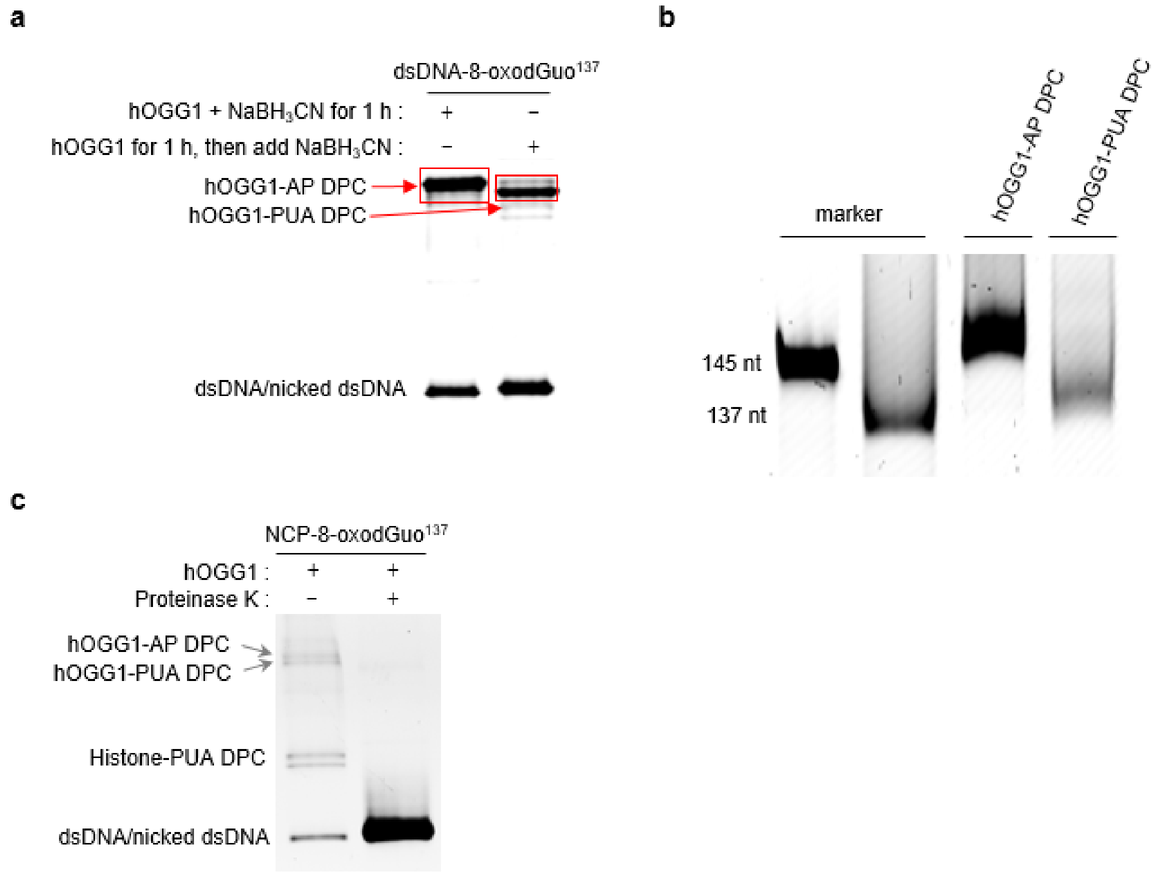

Supplementary Figure S2. Identification of hOGG1-AP DPC and hOGG1-PUA DPC by gel shift assays. **a**) 10% SDS-PAGE analysis of hOGG1-AP DPC and hOGG1-PUA DPC that trapped by $NaBH_3CN$. **b**) 8% denaturing PAGE analysis of hOGG1-AP DPC and hOGG1-PUA DPC. hOGG1-AP DPC and hOGG1-PUA DPC extracted from the SDS-PAGE in **a**) (inside red frames) were digested with proteinase K, and then subjected to 8% denaturing PAGE analysis. **c**) 10% SDS-PAGE analysis of the formation of hOGG1-DNA DPCs and histone-PUA DPCs during the 8-oxodGuo repaired by hOGG1 in NCP-8-oxo-dGuo^{137}.

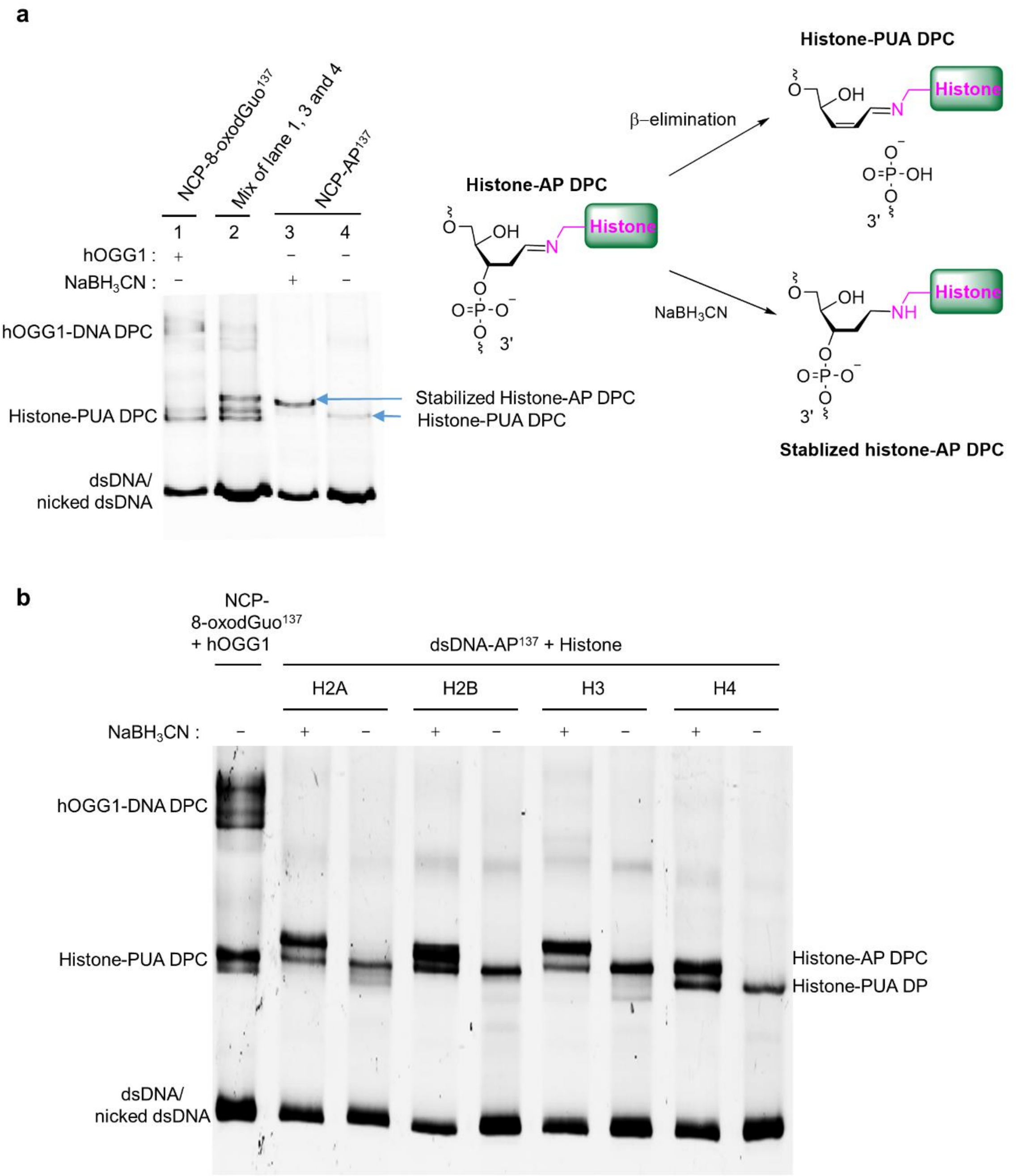

Supplementary Figure S3. Characterization of histone-PUA DPC by gel shift assays. **a**) 10% SDS-PAGE showing the co-migration of histone-PUA DPCs formed during hOGG1 repair of NCP-8-oxodGuo^{137} with authentic histone-DNA DPCs. Authentic histone-AP DPC and histone-PUA DPC were prepared by incubation of NCP-AP^{137} in the presence or absence of $NaBH_3CN$, respectively. **b**) 10% SDS-PAGE showing the migration of histone-PUA DPCs formed during hOGG1 repair of NCP-8-oxodGuo^{137} and different authentic histone-DNA DPCs that are prepared by incubation of dsDNA-AP^{137} with different histones.

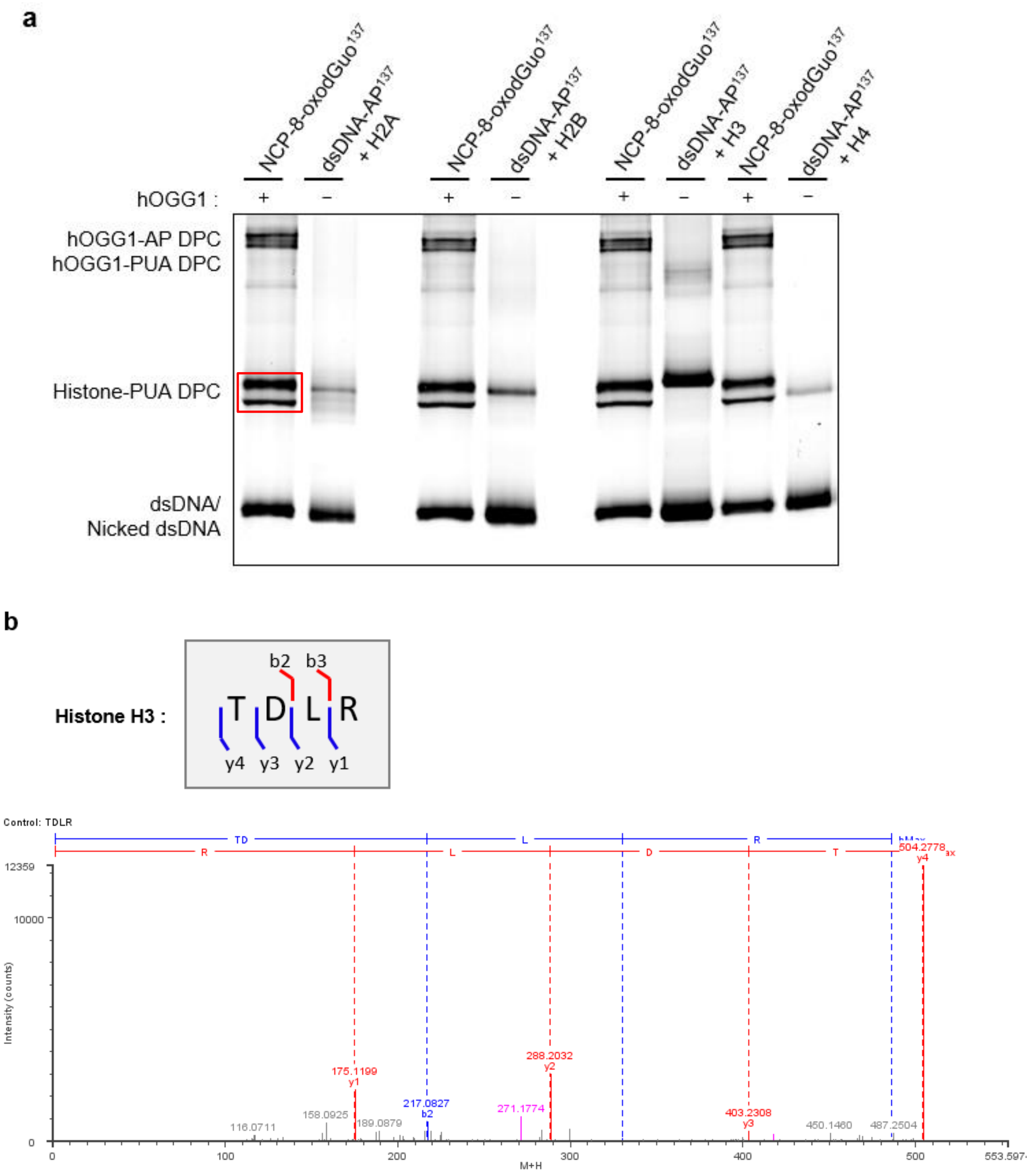
a
NCP-8-oxodGuo137
dsDNA-AP137
+ H2A
+ H2B
+ H3
+ H4
hOGG1 :
hOGG1-AP DPC
hOGG1-PUA DPC
Histone-PUA DPC
dsDNA/
Nicked dsDNA
b
Histone H3 :
T D L R
b2 b3
y4 y3 y2 y1
Control: TDLR
Intensity (counts)
M+H
175.1199 y1
217.0827 b2
271.1774
288.2032 y2
403.2308 y3
504.2778 y4
116.0711
158.0925
189.0879
450.1460
487.2504

c

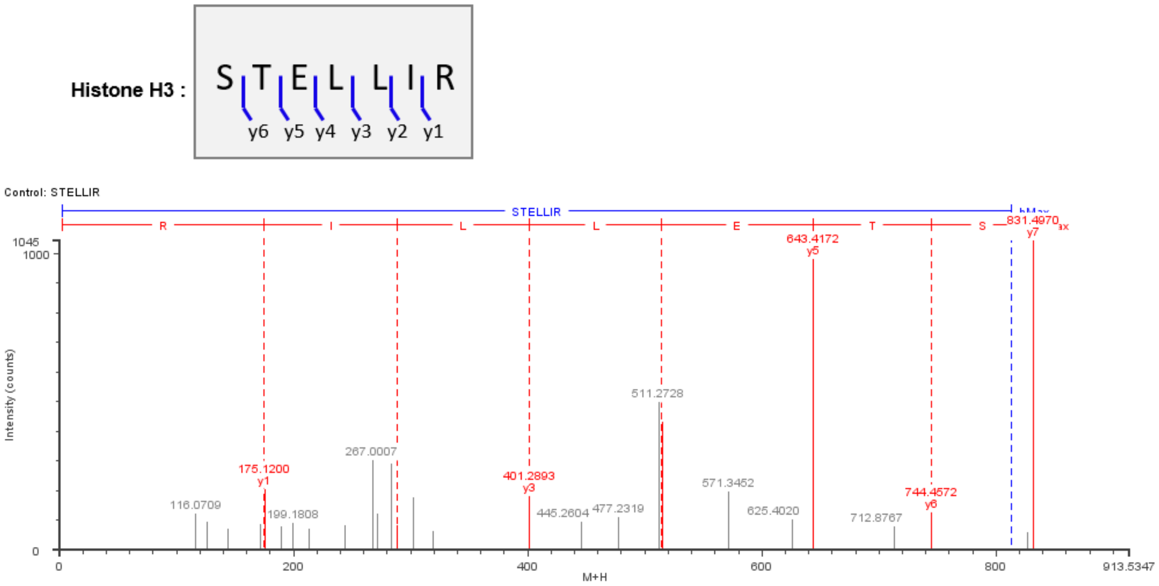

d

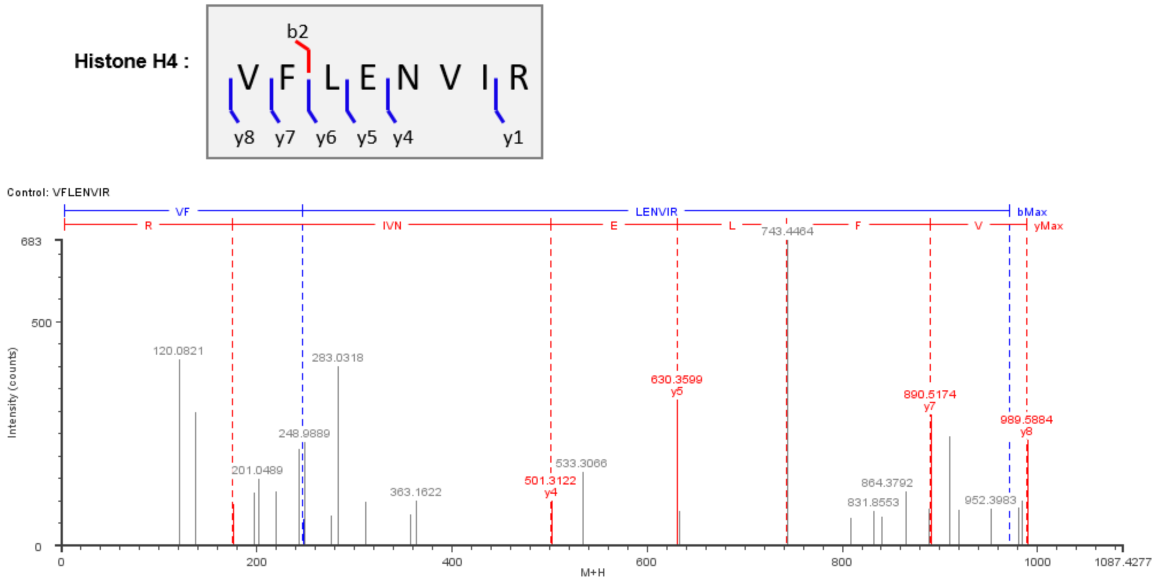

Supplementary Figure S4. Identification of histones included in histone-PUA DPC by gel shift assay and MS/MS analyses. **a**) 10% SDS-PAGE showing the migration of histone-PUA DPC formed during hOGG1 repair of NCP-8-oxodGuo^{137} and different authorized histone-DNA DPCs that are prepared by incubation of dsDNA-AP^{137} with different histones. **b-d**) Peptides identified via MS/MS analyses of the sample that obtained by in gel trypsin digestion of histone-PUA DPCs (Products inside the red frame).

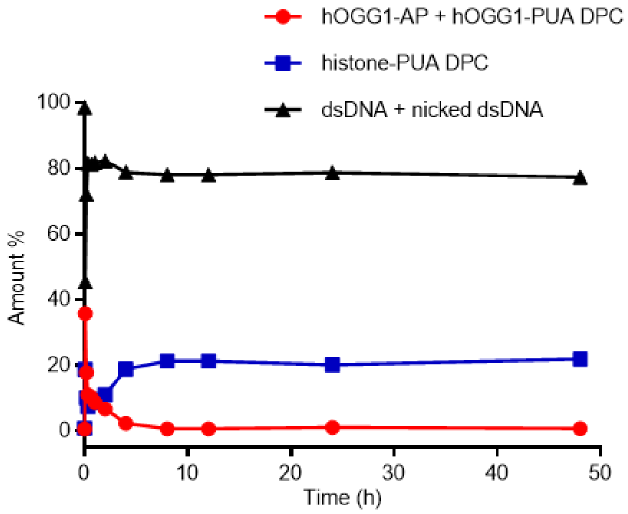

Supplementary Figure S5. Kinetics of formation of histone-PUA DPC during the repair of NCP-8-oxodGuo[137] by hOGG1.

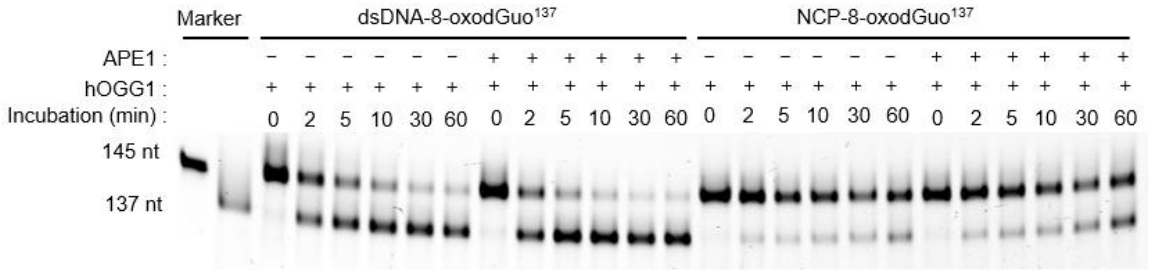

Supplementary Figure S6. 8% denaturing PAGE analysis of kinetics of repair of dsDNA-8-oxodGuo[137] and NCP-8-oxodGuo[137] by hOGG1 in the presence or absence of APE1.

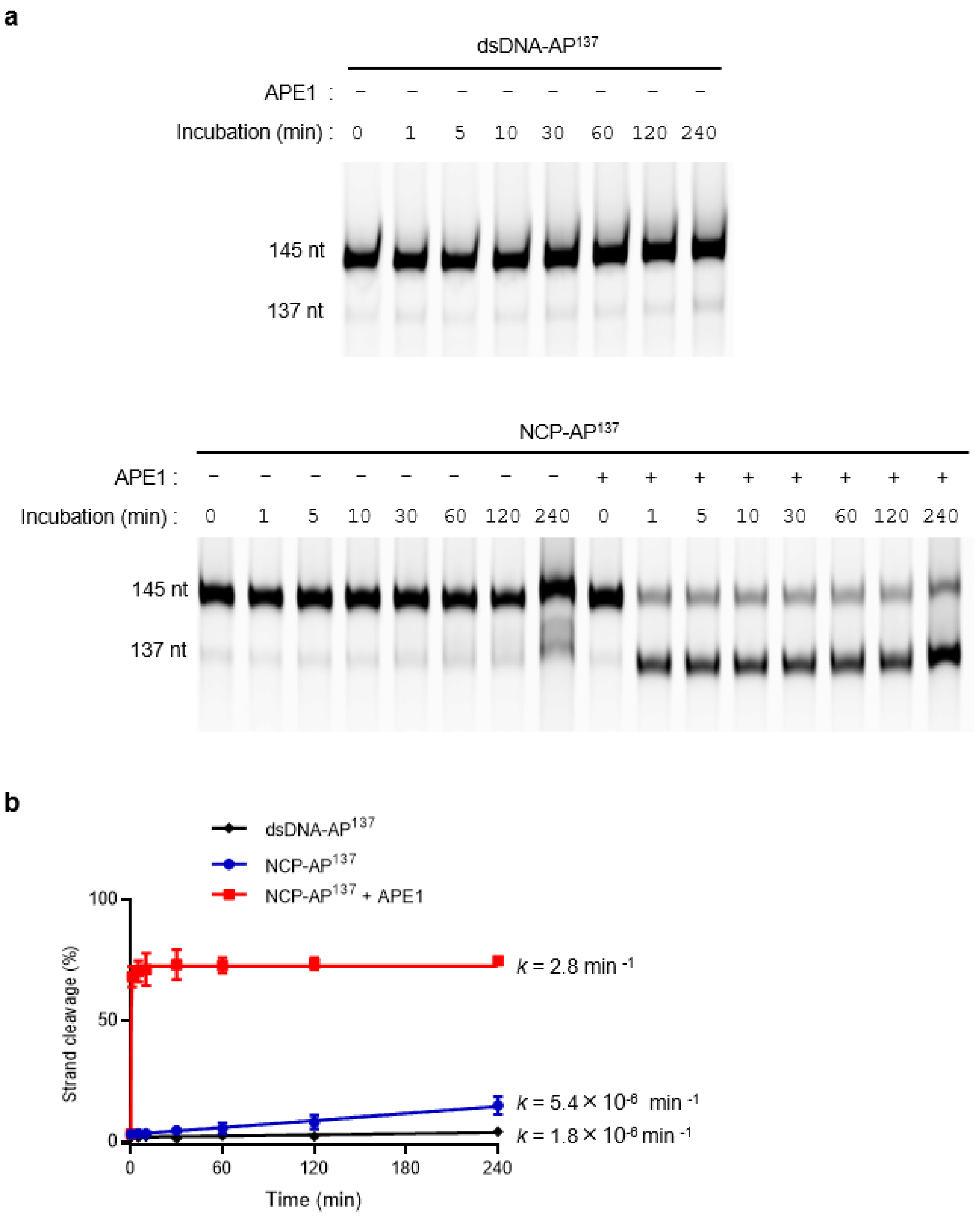

Supplementary Figure S7. **a**) 8% denaturing PAGE analysis of the strand cleavage in dsDNA-AP137 and NCP-AP137 in the presence or absence of APE1. **b**) Kinetics of strand cleavage in dsDNA-AP137 and NCP-AP137 in the presence or absence of APE1.

Reaction conditions: To 38 μL of NCP-AP^{137} solution (8 pmol) in were added 5 μL of 10 × reaction buffer (100 mM HEPES, pH 7.5, 100 mM NaCl, 100 mM $MgCl_2$), 0.5 μL APE1 (6 pmol). The reaction mixture (total volume 50 μL) was incubated at 37 °C.

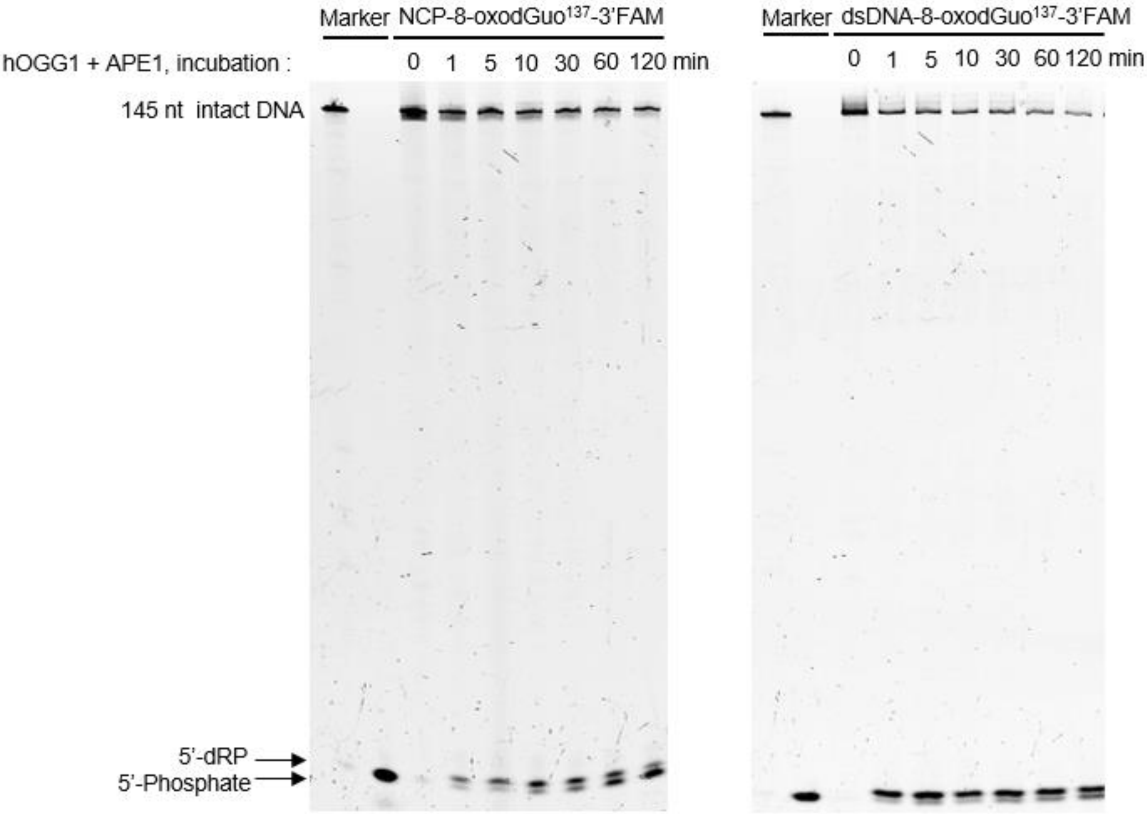

Supplementary Figure S8. 8%/20% two-layer (v/v = 2/5) denaturing PAGE analyses of repair of dsDNA-8-oxodGuo^{137}-3'-FAM and NCP-8-oxodGuo^{137}-3'-FAM by hOGG1 and APE1.

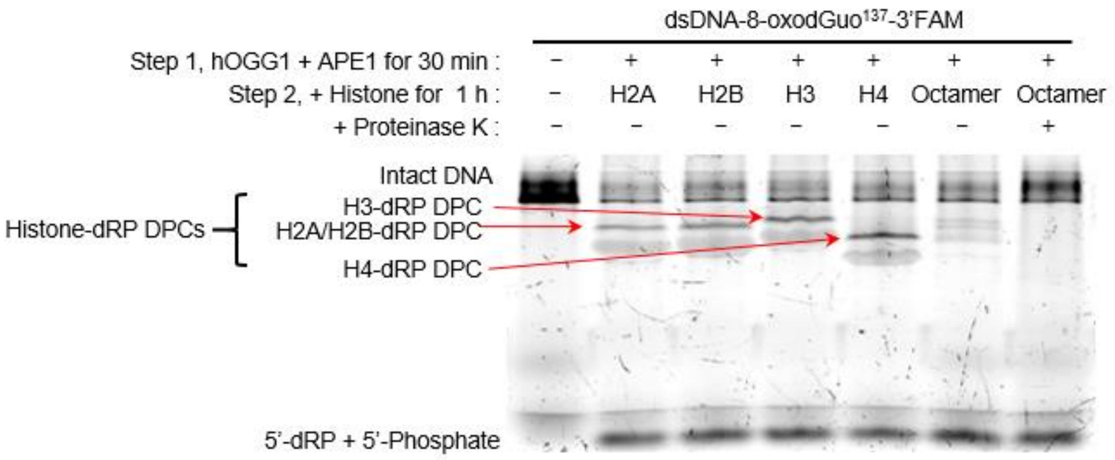

Supplementary Figure S9. 15.5% Tricine-SDS-PAGE analysis of the reactions of dsDNA-8-oxodGuo^{137}-3'FAM with different histones or histone octamer.
